# Detoxification of Aflatoxin B1 by Probiotic Yeasts and Bacteria Isolated From Dairy Products of Iran

**DOI:** 10.34172/apb.2020.060

**Published:** 2020-05-11

**Authors:** Hajar Zolfaghari, Arezou Khezerlou, Ali Ehsani, Ahmad Yari Khosroushahi

**Affiliations:** ^1^Department of Food Science and Technology, Nutrition and Food Science Faculty, Tabriz University of Medical Sciences, Tabriz, Iran.; ^2^Student Research Committee, Department of Food Science and Technology, Tabriz University of Medical Sciences, Tabriz, Iran.; ^3^Food and Drug Safety Research Center, Tabriz University of Medical Sciences, Tabriz, Iran.; ^4^Drug Applied Research Center, Tabriz University of Medical Sciences, Tabriz, Iran.; ^5^Department of Medical Nanotechnology, Faculty of Advanced Medical Science, Tabriz University of Medical Sciences, Tabriz, Iran.

**Keywords:** Aflatoxin B1, Detoxification, ELISA, Food safety, Probiotic bacteria

## Abstract

***Purpose:*** The present study was conducted to assess the ability of probiotic bacteria and yeasts strains to reduce aflatoxin B1 (AFB1) in gastrointestinal simulated conditions. Aflatoxins are potent carcinogenic and immunosuppressive agents. Acute exposure to a high level of aflatoxins leads to aflatoxicosis, which cause rapid death due to liver failure. It is anticipated that consumption of probiotic microorganisms capable of binding aflatoxins can reduce the risk of AFB1 on human health to a certain extent.

***Methods:*** For this purpose, the bacteria (1 × 10^10^ cfu/mL) and yeasts count (2 × 10^8^ cells/mL) and AFB1 concentration (10 ppb) were adjusted. Then, the samples were incubated in the simulated medium, human gastric secretions and small intestine. The concentration of residual AFB1 was determined using enzyme-linked immunosorbent assay (ELISA). The results were statistically analyzed by SPSS 16 software.

***Results:*** The native isolated bacteria and yeasts in the simulated gastrointestinal tract condition showed a significant effect on AFB1 reduction (*P* <0.05). The AFB1 reduction ability of native probiotic microorganisms was strain dependent. The highest binding ability in bacteria belonged to *Lactobacillus rhamnosus* (31.14%) and at yeasts belonged to Saccharomyces cerevisiae (30.46%).

***Conclusion:*** The use of probiotic strains is the appropriate biological method to reduce AFB1 in the human gastrointestinal tract. Probiotic bacteria could help to decrease the harmful effects of AFB1 in humans through enhancing the food safety.

## Introduction

Aflatoxins are considered as secondary metabolites of *Aspergillus flavus* and *Aspergillus parasiticus*, which are thought as one of the most dangerous mycotoxins.^1^ These toxins possess detrimental effects including toxigenic, carcinogenic, mutagenic, teratogenic, and immunosuppressive.^ 2+^ These genotoxic compounds target many organs like kidneys, liver, and immune systems. Their symptoms include: fatty liver, anorexia, diarrhea, vomiting, liver necrosis, and liver cancer. Their impacts on the reproductive system include: delayed testicular development, decreased plasma concentration of testosterone, and decrease in the percentage of live sperm. Also, their immunosuppressive effects include reduced resistance to secondary infections by bacteria, fungi, and parasites. Other related symptoms include encephalopathy and interstitial fibrosis.^3^

Aflatoxins comprise several types of B1, B2, G1, G2, M1 and M2. They can contaminate various types of agricultural and food products such as cereals, oilseeds, spices, tree nuts, and dairy products.^4^ In a large global survey carried out by Taschl and Jenkins,^5^ (covering 18 757 agricultural commodity samples from 72 countries with over 73 000 analyses), the percentage of aflatoxin in Asia (38% of the samples) was shown to be highest compared to other continents. Aflatoxin percentages in agriculture samples of South America, Europe, Middle East, Africa, and North America were reported 23%, 16%, 15%, 11%, and 6%, respectively.

Several strategies have been applied to prevent aflatoxins production or to destroy, to inactivate, or to decrease their bioavailability in contaminated foods. Physical (UV light, heat, or ionizing radiation), chemical (the addition of hydrolytic, chlorinating, or oxidizing agents), or biological methods are used to detoxify aflatoxins.^6,7^ However in the present era, the scientific society tends to use biological approaches instead of chemical and physical methods, due to their some disadvantages such as the loss of nutritional quality and requiring expensive equipments, along with unhealty effects on humans.^8^

One of the most useful biological methods to reduce aflatoxins is the application of probiotic yeasts and bacteria in the diet. Although dairy diets may be associated with dangerous microorganisms,^9^ they are considered as the main source of several types of probiotics.^10^ Since probiotics can bind to aflatoxins into the gastrointestinal tract, thus can prevent the absorption of toxins and reduce the effects of toxins on the animal/human health.^11^ Based on our knowledge, there is scanty published data regarding the ability of probiotics to reduce aflatoxin B1 (AFB1) in gastrointestinal simulation conditions. Native strains of probiotics are adapted to the conditions of their original area and possess the ability to produce good taste and smell in various types of fermented products. Therefore, this study aimed to evaluate the ability of native Iranian probiotics isolated from traditional dairy products to detoxify AFB1 from gastrointestinal simulated conditions.

## Materials and Methods

### Strain of microorganisms (bacteria and yeasts)

In this study, we used indigenous probiotic microorganisms (bacteria and yeasts) isolated from dairy products of Iran (Table 1). All of these strains have been earlier isolated, identified, and categorized for their probiotic properties by Faghfoori et al and Saber et al, and stored in a microbiological collection of the Pharmaceutical Application Research Center laboratory of Tabriz University at 80°C with 25% glycerol.^12,13^

**Table 1:** Twelve indigenous probiotic microorganisms’ strains used in the study and their sources

**Indigenous probiotic strains**	**Source**	**Molecular identification**
*Lactobacillus. Casei*	Isolated from cheese	BH-32
*Lactobacillus. Casei*	Isolated from yogurt	BH-16
*Lactobacillus. plantarom*	Isolated from cheese	BH-7
*Lactobacillus. plantarom*	Isolated from yogurt	BH-14
*Lactobacillus. rhamnosus*	Isolated from cheese	BH-21
*Lactobacillus. rhamnosus*	Isolated from yogurt	BH-17
*Lactobacillus. paracasei*	Isolated from yogurt	B-14
*Saccharomyces cerevisiae*	Isolated from yogurt	AS-27
*Candida krusei*	Isolated from yogurt	AS-29
*Pichia kudriavzevii*	Isolated from yogurt	AS-12
*Candida pseudolambica*	Isolated from cheese	AS-30
*Kluyveromyces marxianus (lactis)*	Isolated from cheese	AS-41

### Preparation of bacterial suspension

Seven indigenous probiotic bacterial strains were used to evaluate their capacity of decreasing AFB1 during simulated gastrointestinal (in vitro system). All bacteria were activated in De Man, Rogosa and Sharpe (MRS) broth and incubated at 37 °C for 24 h. Then, cell culture was centrifuged for 10 minutes at 3000 rpm and the supernatant was separated under sterile conditions. All of cells were washed two times with phosphate-buffered saline (PBS). Finally, with using PBS at pH 7.2, its opacity was obtained by spectrophotometer over a 600 nm wavelength and absorption of about 1 equivalent to 1 × 10^10^ cfu/mL of the cell count of bacteria.^14,15^

### Preparation of yeast suspension

All indigenous probiotic yeast strains were activated in the yeast mold broth (YMB) culture medium at 25 °C for 24 h. Then, cell culture was centrifuged for 10 minutes at 3000 rpm and the supernatant was removed under sterile conditions. Yeast cells were washed twice with PBS. Finally, with using PBS (pH 7.2), its opacity was obtained by spectrophotometer over a 600 nm wavelength and absorption of about 1.170 equivalent to 2 × 10^8^ cells/mL of the cell count of yeast.^16^

### Preparation of the stock solution of Aflatoxin B1

The AFB1 required for this study was purchased from Sigma Company (Sigma, St. Louis, MO, USA, CAS number: A6636) in the form of a vial having one g toxin powder. This powder was suspended in benzene–acetonitrile (97:3, v/v). PBS (pH 7.2) was prepared and benzene–acetonitrile was separated with a rotary evaporator for 10 min at 80°C and final concentration of AFB1 (10 ppm) was made through dilution with PBS. The stock solution was stored at 4°C in an amber glass until uses.^17^

### Preparation of digestive fluids (Stomach fluid, intestinal fluid)

A suspension of the simulated stomach fluid containing KCl 2.2 g/L, NaCl 6.2 g/L, CaCl_2_ 0.22 g/L, NaHCO_3_ 1.2 g/L, pepsin 0.3% and a suspension of the simulated intestinal fluid containing KCl 0.6 g / L, NaCl 5 g/L, CaCl_2_ 0.3 g/L, 0.45% bile salts, 0.1% pancreatin was prepared. Then, the pH of the stomach fluid decreased to 2.5 with the addition of a 0.1 N hydrochloric acid (HCl) solution and pH of intestinal fluid increased to 7.5 with a 0.1 N sodium hydroxide (NaOH) solution. Finally both suspensions were filtered with a 0.22 μM filter.^18^

### Culturing the samples in simulated gastrointestinal conditions

**Figure 1:**
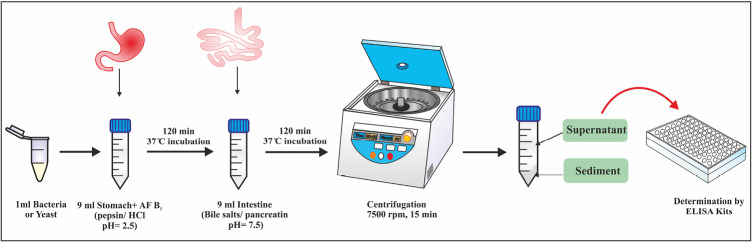
Schematic representation of in vitro simulated gastrointestinal conditions to assay the reduction of aflatoxin B1.

Culturing the samples in simulated gastrointestinal conditions was included two sequential steps; First, to simulate the stomach conditions, 1 mL of the active culture of each probiotic strain (10^10^ cfu/mL) was added to the 9 ml simulated stomach fluid (pepsin / HCl, pH 2.5) contaminated with 10 ppb AFB1 toxin. Then after vortexing for fifteen seconds, it was incubated at 37°C for 120 minutes. In the next step, 1ml simulated stomach fluid containing the AFB1 toxin and the active culture of each probiotic strain was added to the 9 mL simulated intestine fluid (bile salts/pancreatin pH 7.5). Then, it was incubated at 37°C for 120 minutes, to simulate the intestine conditions. After incubation, the samples were transferred to microtubes and centrifuged at 7500 rpm for 15 minutes and bacteria were precipitated. Then, the supernatant solution was removed and to ensure more separation of all bacteria, the centrifuge was purified again under the same conditions as before and its supernatant was removed. After that, the ELISA method was used to determine the amount of AFB1 remaining in the test solution and compared with the amount of AFB1 available in the control solution, which lacked bacteria or yeasts but contained the same amount of AFB1. The decrease in the amount of AFB1 in the test solution was compared to the control solution, indicating the ability of the bacteria and yeasts strains to absorb toxins and remove them from the solution. AFB1 ELISA kit (r-Biopharm; Darmstadt, Germany) was used and the method employed was based on the competitive ELISA. The schematic diagram of Figure 1 shows the procedure steps, briefly.

### Quantitative analysis of Aflatoxin B1 with ELISA

The quantitative analysis of AFB1 was determined by competitive enzyme immunoassay using the ELISA test kit. To calculate the AFB1 concentration, a standard curve was prepared using obtained AFB1 standard solutions at concentrations of 0, 1, 5, 10, 20, and 50 ng/mL. Fifty μL of AFB1 standards and 50 μL of prepared samples were added into separate duplicated wells of micro-titer plate precoated with AFB1 antibodies. Subsequently, 50 μL of the enzyme conjugate along with 50 μL of the anti-aflatoxin antibody solution were included to the each well. Plate was shaken and incubated at room temperature for 30 minutes. After the washing step, 100 μL of substrate/chromogen was added to the each well and incubated further at room temperature for 15 minutes. The process was hindered through incorporation of 100 μL of the stop solution and the absorbance was measured at 450 nm in ELISA plate reader.^19^ The concentration of AFB1 in standard and samples was measured using the following formula.

$$\%Absorbance=\frac{Absorbance\;value\;of\;sample\;or\;s\tan dard\;solution}{Absorbance\;value\;of\;zero\;s\tan dard\;solution}\times 100$$

### Statistical analysis

The statistical package for the social sciences (SPSS Inc., Chicago, IL, USA version 16.0) was utilized to analyze the data. One-way ANOVA and Duncan post hoc test were utilized for the variance analysis between all samples and comparison of multiple means, respectively. Statistical significance was defined as a value of *P* <0.05. The quantitative statistics were presented as mean ± SD. All of the experiments were done in triplicate. Diagrams were designed with Excel software.

## Results and Discussion

**Figure 2:**
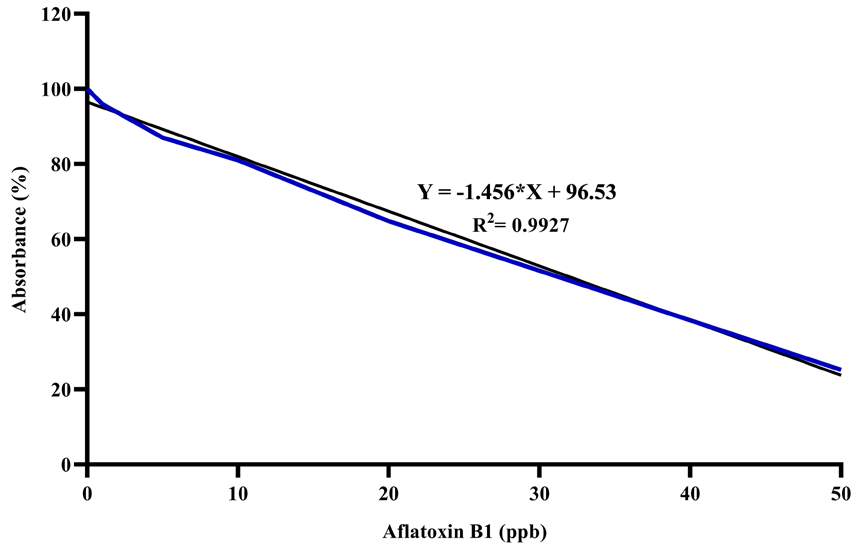
Standard curve for Aflatoxin B1 by competitive ELISA.

**Figure 3:**
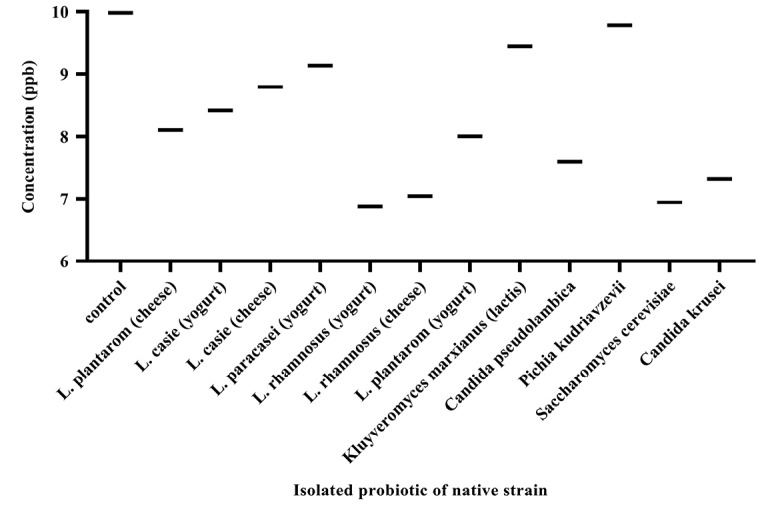
Aflatoxin B1 concentrations in the binding assays with native probiotic microorganisms.

The standard curve for AFB1 was drawn at 0-50 ppb range by the Excel software (Figure 2). The AFB1 concentration of each sample was determined at ppb scale according to the absorption rate based on the standard curve. Figure 3 illustrates concentrations of AFB1 in the binding assays with native probiotic microorganisms in the gastrointestinal simulated condition.

**Figure 4:**
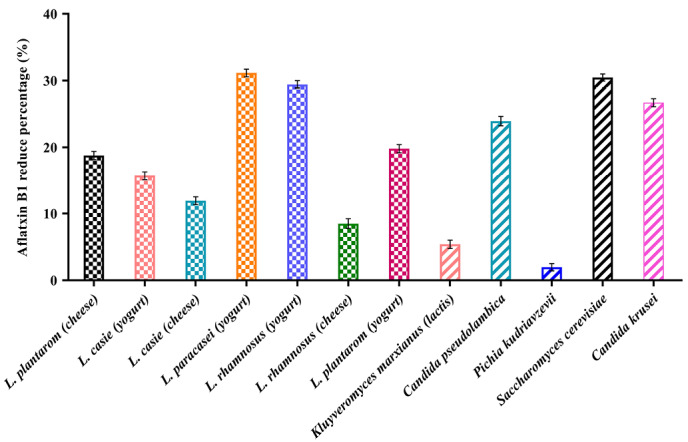
Comparison of reduction percentage of Aflatoxin B1 by native probiotic bacteria and yeasts.

Analysis of the data (Figure 4) showed that the percentage of decreased aflatoxin was significant in all samples (*P* <0.05). The AFB1 binding ability was variable in different strains isolated. The percentage varies between 1.96% and 31.15%. Bacteria and yeasts isolates obtained from dairy products of Iran showed an ability to reduce AFB1.

The binding ability of native probiotics *Lactobacillus* was observed to be strain variant and ranged from 8.38% to 31.14%. The highest binding of AFB1 was obtained by *L. rhamnosus* isolates from yogurt (31.14%), *L. rhamnosus* isolates from cheese (29.43%), *L. plantarum* isolates from yogurt (19.82%), *L. plantarum* isolates from cheese (18.78%), respectively. In contrast, *L. paracasei* isolates from yogurt showed the least reduction of 8.48 %.

Concerning the yeasts, the highest binding of AFB1 was obtained by *Saccharomyces cerevisiae* isolates from yogurt (30.46%), *Candida krusei* (26.69%) and *Candida pseudolambica* isolates from cheese (23.93%), and in contrast, *Kluyveromyces marxianus* (lactis) isolates from cheese (5.40%) and *Pichia kudriavzevii* isolates from yogurt (1.96%) showed the least reduction. Our study was in line with studies of Bovo, Franco.^20^

Two main mechanisms have been known by the biological system (use of bacteria and yeast) to deal with AFB1, called enzymatic and absorption mechanisms.^21,22^ The enzymatic one is based on the degradation of mycotoxins by two different enzymes. First, a nicotinamide adenine dinucleotide phosphate (NADPH) dependent enzyme, named 17-hydroxy-steroid dehydrogenase, which transforms AFB1 to aflatoxical through the addition of hydroxyl groups to the double bond of dihydrofuran ring. This product then is excreted via urine and feces. The second way is attributed to the function of carboxypeptidase A, an oxidative enzyme. This enzyme cleaves the, β-moiety ester and bisfuran ring of AFB1 to the degraded products such as aflatoxical, aflatoxin B2a, AFD1, AFD2; AFO, AFB2a, AFD1, AFD2.^23-25^ Another mechanism is the absorption of AFB1 to the surface of probiotic bacteria and yeasts. To reduce AFB1 through absorbing on yeast surface, the involving mechanism is the trapping of AFB1 by the β-D-glucans component of yeast cell wall. AFB1 is trapped in the single helix of (1→3)-β-D-glucan chains and the branched (1→6)-β-D-glucan chains, thereby keeping the toxin inside the helix structure.^26^

Our study showed that *L. rhamnosus* (isolated from yogurt or cheese) has the highest percentage of aflatoxin removal, which is compatible with studies of Zinedine, Faid^17^ and Haskard, El-Nezami.^27^ Unfortunately, there is not a clear reason for the superior ability of *L. rhamnosus* to remove AFB1 in comparison with other lactic acid bacteria. But, the stronger removal of aflatoxin by non-viable *L. rhamnosus* compared to viable cells indicates that the adsorption of aflatoxin on the surface of cell wall is the dominant mechanism of toxin elimination by these organisms.^28^

It is thought that certain binding sites of cell wall peptidoglycan (PG) containing protein and carbohydrate components participate in the adsorption of aflatoxins. However, many sections such as lipopolysaccharide, lipoteichoic acid, N-linked glycans, and proteins attached non-covalently to the surface of *L. rhamnosus* have been exhibited not to have any effect on the adsorption of aflatoxins.^15,29^ On the other hand, it is assumed that the adsorption of aflatoxins to PG is carried out by non-covalent connections such as hydrogen bonds or van der waals interactions, since the washing of *L. rhamnosus* strains containing adsorbed aflatoxins by the phosphate-buffered saline solution leads to the release of a high amount of adsorbed toxins.^30,31^

It has been also shown that *L. rhamnosus* exposed to acidic environments (non-viable cells) attracts aflatoxins more efficient than the viable bacteria. It is guested that the increase in hydrophobic sites on the surface of PG, due to the denaturation of protein as well as the separation of manan carbohydrates and likewise the generation of many pores in the PG structure, results in the remove of more aflatoxins.^32,33^

Similar to bacteria, there is not a certain reason for the higher ability of *S. cerevisiae* to remove AFB1 compared to other yeasts. The dominant mechanism, by which toxins are bound to *S. cerevisiae* is attributed to the formation of a reversible complex between the yeast cell wall and the toxin.^34^ Also, it has been displayed that (1→3) β-D-glucan is the most efficient binder on the surface of *S. cerevisiae* cell wall.^35^ The cell wall of this yeast has been mostly made up of polysaccharides (80-90%). An inside layer composed of β-D glucans is responsible for the mechanical resistance of cell wall. This layer is an interwoven network of polymerized (1→3) β-D-glucans, which are branched off as (1→6) β-D-glucans. These β-D-glucans can form helical conformations adopted from triple or single helix polysaccharide chains, constructing a fibrillary structure.^36,37^

## Conclusion

Findings of the current study showed that probiotic strains isolated from traditional dairy products have the potential to remove AFB1. The highest binding ability in bacteria belonged to *Lactobacillus rhamnosus* (31.14%), and at yeasts, belonged to *S. cerevisiae* (30.46%). Variations and differences in reduction abilities among strains suggest that different binding sites could be present in different strains. The results can indicate that, if foods are enriched with specific indigenous probiotic microorganisms, they would reduce the toxicity risk of aflatoxins in foodstuffs.

## Ethical Issues

No ethical issues for this work.

## Conflict of Interest

No conflict of interest with any organization, reviewers and authors for this work.

## Acknowledgments

The authors extend their gratitude to the Vice Chancellor of Research and Technology at the Tabriz University of Medical Sciences (Tabriz, Iran) for his financial support. This study was financially supported by the Tabriz University of Medical Sciences. The results of this article were extracted from the M.S. thesis of Hajar Zolfaghari, which is registered at the Tabriz University of Medical Sciences.
